# Menthol Targeting AMPK Alleviates the Inflammatory Response of Bovine Mammary Epithelial Cells and Restores the Synthesis of Milk Fat and Milk Protein

**DOI:** 10.3389/fimmu.2021.782989

**Published:** 2021-12-22

**Authors:** Songqi Liu, Wenjin Guo, Yuxi Jia, Bojian Ye, Shu Liu, Shoupeng Fu, Juxiong Liu, Guiqiu Hu

**Affiliations:** ^1^ College of Veterinary Medicine, Jilin University, Changchun, China; ^2^ Department of Orthopedics, The Second Hospital of Jilin University, Changchun, China; ^3^ Application Demonstration Center of Precision Medicine Molecular Diagnosis, The Second Hospital of Jilin University, Changchun, China

**Keywords:** mastitis, menthol, BMECs, AMPK, autophagy

## Abstract

Mastitis is one of the most serious diseases that causes losses in the dairy industry, seriously impairing milk production and milk quality, and even affecting human health. Menthol is a cyclic monoterpene compound obtained from the stem and leaves of peppermint, which has a variety of biological activities, including anti-inflammatory and antioxidant activity. The purpose of this study was to investigate the preventive effect of menthol on the lipopolysaccharide-induced inflammatory response in primary bovine mammary gland epithelial cells (BMECs) and its anti-inflammatory mechanism. First, BMECs were isolated and amplified from the udders of Holstein cows by enzymatic hydrolysis. BMECs were treated with menthol (10, 50, 100, 200 μM) for 1h, followed by lipopolysaccharide (5μg/ml) for 12 h. Lipopolysaccharide treatment upregulated the protein levels of cyclooxygenase-2 (COX-2) and inducible nitric oxide synthase (INOS) and the mRNA abundance of tumor necrosis factor α (TNF-α), interleukin-6 (IL-6), and interleukin-1β (IL-1β), while menthol was able to inhibit this effect. The inhibitory effect of menthol on proinflammatory factors was significantly reduced when autophagy was blocked using 3-Methyladenine (5μg/ml), an inhibitor of autophagy. Furthermore, lipopolysaccharide treatment reduced the expression levels of milk lipids and milk proteins, which were inhibited by menthol. In addition, menthol (200 μM) treatment was able to significantly upregulate the expression level of autophagy-related protein LC3B, downregulate the expression level of P62, promote the expression abundance of autophagy-related gene mRNA, and enhance significantly enhance autophagic flux. Interestingly, treatment of BMECs with menthol (200 μM) promoted the phosphorylation of AMP-activated protein kinase (AMPK) and unc-51 like kinase 1 (ULK1) and increased the nuclear localization of nuclear factor-E2 associated factor 2 (Nrf-2). When the AMPK pathway was blocked using compound C (10μg/ml), an inhibitor of AMPK, autophagy was significantly inhibited. Autophagy levels were significantly decreased after blocking the Nrf-2 pathway using ML385 (5μg/ml), an inhibitor of Nrf-2. Overall, the data suggest that menthol activates the AMPK-ULK1 pathway to initiate the onset of autophagy and maintains the level of autophagy through the AMPK-Nrf-2 pathway. In conclusion, the findings suggest that menthol may alleviate the inflammatory response in BMECs *via* the AMPK/ULK1/Nrf-2/autophagy pathway.

## Introduction

Milk has made a significant contribution to ensuring human nutrition and health, and it also has an important pulling effect on the industrial chain and circular economy (1). Therefore, the dairy industry has become an important symbol to evaluate the degree of development of the livestock industry of a country. Mastitis in dairy cows is well known to be one of the most serious diseases causing losses in the dairy industry, which can seriously damage milk production and milk quality (2, 3), and even affect human health (4). Mastitis in dairy cows is a complex inflammatory response caused by conditionally pathogenic bacteria, and studies have shown that *Escherichia coli (E. coli)*, a common gram-negative bacterium, has a very important role in the occurrence and development of mastitis (5). lipopolysaccharide, as the main component of *E. coli* endotoxin, can cause a severe immune response and promote the development and progression of inflammation (6).

Bovine mammary epithelial cells (BMECs) are the first line of defense against bacterial attack in the external environment (7). When mastitis occurs in cows, mammary epithelial cells or macrophages in cows are able to generate an immune response under LPS stimulation and release large amounts of proinflammatory factors that destroy BMECs, causing damage to mammary tissue (8). The bacterial endotoxin-induced inflammatory response in BMECs can significantly affect milk yield and lead to a reduction in milk protein and milk lipid expression levels per unit volume of milk (3, 9). Therefore, it is important to control the inflammatory response of BMECS during the treatment of mastitis.

It is well known that the inflammatory response is a self-protective mechanism of the body, that has the function of removing pathogenic bacteria, eliminating bacterial toxins and repairing damaged tissues (10). When the autoimmunity of the body is able to clear and kill pathogenic bacteria and repair the damage, mastitis will recover on its own; otherwise, the inflammatory response will become more intense, not only damaging the mammary tissue and reducing milk production; but also even endangering the life of cow (11). Several studies have shown that the inflammatory response is closely linked to autophagy, that autophagy plays a key role in maintaining inflammatory homeostasis, and that enhancing autophagy can reduce damage to the inflammatory response (12–14). It has been shown that autophagy can protect cells from damage by excessive and persistent inflammatory responses by removing damaged organelles (e.g., mitochondria) or intracellular pathogenic microorganisms and molecules that inhibit proinflammatory factors in a variety of ways (15, 16). Autophagy is an important process for maintaining intracellular homeostasis and is regulated by multiple autophagy-related pathways (15, 17). Our previous study also showed that Schizandrin A was able to directly activate autophagy through the AMPK-ULK1 pathway and inhibit LPS-induced mouse mastitis (18). Niacin can regulate autophagy and thus reduce the inflammatory response of BMECs by activating GPR109A (19).

Menthol is an aromatic compound with high anti-inflammatory activity, which is a cyclic terpene alcohol obtained from the stem and leaves of peppermint (20, 21). Menthol has long been studied in medicine (22), and today it is widely used in applications, ointments and tablets for the relief of pain and irritation of the airways and skin (23). Moreover, menthol has shown strong anti-inflammatory and antioxidant activities in various *in vitro* and *in vivo* models according to relevant studies (20, 24). At the same time, menthol is extremely inexpensive to obtain, and its large-scale application does not significantly increase the cost of mastitis treatment. However, it is not clear whether menthol can alleviate LPS-induced mammary inflammation. Therefore, we designed the following experiments to investigate the role and mechanism of menthol in the LPS-induced mastitis model of BMECs.

## Materials and Methods

### Reagents

Menthol purchased (a colorless crystal, purity≥98%) from Shanghai Yuanye Biotechnology. Lipopolysaccharides (LPS), phenylmethanesulfonylfluoride (PMSF), Compound C (CC) and dimenthy sulfoxide (DMSO) purchased from Sigma-Aldrich. 3-methyladenine (3-MA) and ML385 were purchased from Selleck Chem.

### Antibodies

AMPK (1:1000, CAT:5823S), p-AMPK(1:1000,CAT:2535S), and p-ULK1(1:1000,CAT:5869T00) purchased from Cell Signaling Technology. ULK1(1:1000,CAT:20986-I-AP), P62(1:1000,CAT:66184-2-2g), Nrf-2(western blot:1:1000, IF:1:150, CAT:16396-1-AP), LC3B(1:1000, CAT:18725-1-AP) and β-Actin (1:2000, CAT:20536-1-AP) purchased from Proteintech. Heme Oxygenase-1(HO-1) (1:1000, CAT : GK284419-11), COX-2(1:1000, CAT:ab62331) and INOS (1:1000, CAT:ab1532) purchased from Abcam. The secondary antibodies used in this study, including Alexa Fluor488 donkey anti-rabbit or mouse IgG (H+L) high cross-sorbent secondary antibodies, were purchased from Life Technologies (Carlsbad, CA, USA). Enzyme-labeled goat anti-mouse and goat anti-rabbit secondary antibodies were purchased from Bosterbio (Pleasanton, CA, USA).

### Isolation and Culture of BMECs

The isolation and culture of bovine mammary epithelial cells from dairy cows, was described by Huang et al. (25). Briefly, mammary tissue from lactating Holstein cows was collected aseptically from a dairy slaughterhouse and placed in 50 ml sterile centrifuge tubes that included ice-cold Ham’s F12 medium with 1 × antibiotic/antifungal drug (100 U/mL penicillin, 100 μg/mL streptomycin, 0.25 μg/mL amphotericin B, 50 μg/mL gentamicin) and transported to the laboratory. The visible fat, connective tissue and blood vessels were first removed, and the breast tissue was cut into small pieces of approximately 1 cm**^3^** using surgical scissors and then washed 5 times with Ham’s F12 medium supplemented with 1 × antibiotics/antimycotics to remove milk and blood. Chopped tissues were subjected to enzymatic digestion in DMEM/F12, a medium containing 300 U/ml collagenase type 3, 400 U/ml hyaluronidase and 1 mg/ml deoxyribonuclease I, supplemented with 1 × antibiotic/antifungal drug, and shaken continuously (80 rpm) for 4 h at 37°C. Tissue digests were filtered through a 200-μm sieve and then centrifuged at 80 × g for 30 seconds at room temperature. The resulting precipitate was enriched with mammary epithelial organoid cells (glandular vesicles). The precipitate was resuspended in BMEC growth medium for the growth of mammary epithelial cells. BMECs growth medium consisted of 1:1 DMEM/F12:MCDB170 (Item no.: M2162, American Biologicals, Salem, MA), 0.25% (v/v) fetal bovine serum, 0.1% (w/v) albumin II, 7.5 µg/mL bovine insulin, 0.3 µg/mL hydrocortisone, 5 ng/mL recombinant human epidermal growth factor, 2.5 µg/mL bovine apolipoprotein-transferrin, 5 µM isoprenaline, and 5 pM 3,3′,5-triiodine-1. Primary mammary epithelial cells from glandular follicle growth were passaged once for amplification and then frozen for storage. Unless otherwise stated, cell culture reagents were purchased from Sigma Aldrich or Thermo Fisher Scientific.

### Separation of Nucleus and Cytoplasm

We inoculated BMECs in the cell culture dishes (705001,NEST,Wuxi,China) and treated them with reagents (menthol,CC and ML385) when the cells reached about 80%. After the cells were washed twice with cold PBS after the above treatment was completed (26), the separation of cytoplasmic and cytoplasmic proteins was performed according to the instructions of the Cytoplasmic and Cytoplasmic Protein Extraction Kit (CAT: P20027, Beyotime).

### Cell Counting Kit-8 Assay

To investigate the effect of menthol on the activity of BMECs, we measured the activity of BMECs using Cell Counting Kit-8 (CCk-8) (Saint-Bio, Shanghai, China). BMECs were cultured in 96-well plates for 12h. After 3h starvation of the cells using serum-free medium, the cells were treated with DMSO (solvent control group) and different concentrations of drugs (10µM,50µM,100µM,200µM,300 µM,500 µM,1000 µM of menthol) for 1h, followed by 12h treatment with LPS (5µg/ml). Subsequently, 10 µl of CCK-8 solution was added to each well and incubated for 1 h. Finally, the absorbance of each well at 450 nm was measured.

### Quantitative Real-Time PCR (qRT-PCR)

Total RNA was extracted from BMECs using TRIzol (Life Technologies) and then reverse transcribed into cDNA using a ReverAid First Strand cDNA Synthesis Kit (Thermo Fisher Scientific) to reverse transcribe into cDNA. In this experiment, 20 μl system was used, and 1 μl of forward primer, 1 μl of reverse primer, 8 μl of cDNA template after 10-fold dilution, and PCR mix fluorescent dye 10μl were added into the real-time qRT-PCR instrument for detection. qRT-PCR was performed with a SYBR Green QuantiTect qRT-PCR Kit (Roche). The annealing temperature in the program was adjusted appropriately according to the Tm values of the different primers. The primers were designed and synthesized by Biotech, and the sequence pieces are listed in **Table 1** (27). In this experiment, β-actin was used as an internal reference gene, and the mRNA expression levels of the relevant target genes were calculated by the 2^-ΔΔCt^ method.

**Table 1:** The primer sequences for β-actin, IL-1β, IL-6, TNF-α, LC3B, P62, etc.

Gene	Sequences	Length (bp)	Tm (°C)
β-actin	(F)5′-TCACCAACTGGGACGACA-3′	205	58
	(R)5′-GCATACAGGGACAGCACA-3′		
IL-6	(F)5′-ATGCTTCCAATCTGGGTTC-3′	269	55
	(R)5′-TGAGGATAATCTTTGCGTTC-3′		
IL-1β	(F)5′-AGGTGGTGTCGGTCATCGT-3′	195	60
	(R)5′-GCTCTCTGTCCTGGAGTTTGC-3′		
TNF-α	(F)5′-ACGGGCTTTACCTCATCTACTC-3′	140	60
	(R)5′-GCTCTTGATGGCAGACAGG-3′		
ATG4B	(F)5′-AGGTGGACGCAGCGGAAGAG-3′	183	63
	(R)5′-GACAGCCAGCTTCTTGAGGACTTG-3′		
ATG4D	(F)5′-GACAGCCAGCTTCTTGAGGACTTG-3′	205	63
	(R)5′-GTAGCCGATGAAGTACAGCGAGTG-3′		
ATG5	(F)5′-AGCATCATCCCGCAACCAAC-3′	191	62
	(R)5′-GACCAGCCCTAGTGCCCTTA-3		
ATG12	(F)5′-AAGATGGCTGAGGAGCAGGAGTC-3′	81	63
	(R)5′-GGAGACCTCGGTAGGCACTTCAG-3′		
Beclin-1	(F)5′-TGCACAGACACTCTCCTAGACCAG-3′	167	63
	(R)5′-ATCAGCCTCTCCTCCTCTAATGCC-3′		
ULK1	(F)5′-GCATCGGCACCATCGTGTACC-3′	105	63
	(R)5′-GGACCAGCGTCTTGTTCTTCTCG-3′		
P62	(F)5′-GTGATCTGTGACGGCTGTAACGG-3′	85	63
	(R)5′-AGGCGGAGCATAGGTCGTAGTC-3′		
LC3B	(F)5′-GCAGGCCACCGTTCACTCTTG-3′	143	63
	(R)5′-ATGCAGCAGGAAGAGCAGATTGG-3′		

### Primer Sequences

The primer sequences for β-actin, IL-1β, IL-6, TNF-α, LC3B, P62, etc. (27) are shown in **Table 1**.

### Immunofluorescence

The treated BMECs were washed 3 times with PBS for 5 min/wash, and the BMECs were fixed with immunostaining fixative (P0098, Beyotime) for 10 min at room temperature and washed 3 times with PBS for 5 min/wash. Cells were incubated in PBS containing 5% donkey serum for 3 h at room temperature, washed 3 times with PBS for 5min/wash, and incubated overnight at 4°C with primary antibody diluted in PBS containing 5% donkey serum. The samples were incubated overnight, washed 3 times with PBS for 5 min/wash and incubated for 1 h at room temperature with secondary antibody diluted in PBS containing 5% donkey serum. Finally, the cells were washed 3 times with PBS for 5 min/wash and stained with DAPI for 5 min. Images of stained cell sections were obtained by scanning with immunofluorescence microscopy(Olympus, Japan).

### Western Blotting Analysis

Total protein was separated from the treated mammary epithelial cells using RIPA lysis buffer (Beyotime); and then centrifuged at 4°C; and 12,000 g for 15 min. The supernatant containing the total protein was collected. Similarly, nuclear proteins were obtained using the Nucleus and Cytoplasm Protein Extraction Kit (Beyotime). The protein concentration was subsequently determined using a BCA protein concentration assay kit (Beyotime). Protein samples were added to 4%-15% SDS-PAGE; and separated in a 110 V electric field for 90 min, and then the separated proteins were transferred to PVDF membranes (Millipore) at 75 V; for 60 min. The PVDF membranes containing proteins were closed at room temperature for 2 h in TBS-T containing 5% skimmed milk. The primary antibodies were then incubated overnight at 4°C, and each primary antibody was incubated using a TBS-T solution containing 5% bovine serum protein(Gentihold). After the primary antibody incubation was completed, the membrane was washed 5 times with TBS-T, and then the PVDF membrane was incubated for 1 h at room temperature (1:5000 dilution) with the corresponding species of enzyme-labeled secondary antibody (Bosterbio). Finally, the immunoreactive blots were visualized with an enhanced chemiluminescent substrate (Beyotime).

### BODIPY Staining

Cells were treated with 200 μM menthol and/or 5 μg/ml LPS, washed 3 times with PBS for 5 min/wash, fixed with immunostaining fixative (P0098, Beyotime,Shanghai,China) for 20 min, and washed 3 times with PBS for 5 min/wash. Then, the lipid droplets were stained with the lipid dye BODIPY493/503 for 20 min (green) in a dark room; and washed 3 times with PBS for 5 min/wash, and the distribution of lipid droplets was observed under a fluorescence microscope (Olympus,Japan) after the addition of DAPI (28).

### Construction of Plasmids

BMECs were inoculated in 24-well plates with microscope slides and transfected with mRFP-GFP-LC3 (gift from the Pathology Laboratory, College of Veterinary Medicine, Jilin University) for 24 hours using LiopFiter (HABIO, Qingdao,China). After the indicated treatments, the BMECs were washed 3 times with PBS for 5 min/wash and fixed with immunostaining fixative(P0098, Beyotime) for 10 min at room temperature and 3 times with PBS for 5 min/wash. All cell images were obtained using an inverted confocal microscope (Olympus, Japan).

### Statistical Analysis

In this study, all data analysis work was performed using GraphPad Prism (La Jolla). The final results are expressed as the mean ± SEM. Statistical differences between groups were analyzed by one-way ANOVA followed by Tukey’s multiple comparisons. The data significance analysis uses one-way analysis and uses *, **, *** and **** to indicate differences between groups. A value of *p < 0.05 was considered significant, **p < 0.01 was considered highly significant, and ns was consider no difference. Error bars are the SEM.

## Result

### Menthol Attenuates the LPS-Induced Inflammatory Response in BMECs

**Figure 1:**
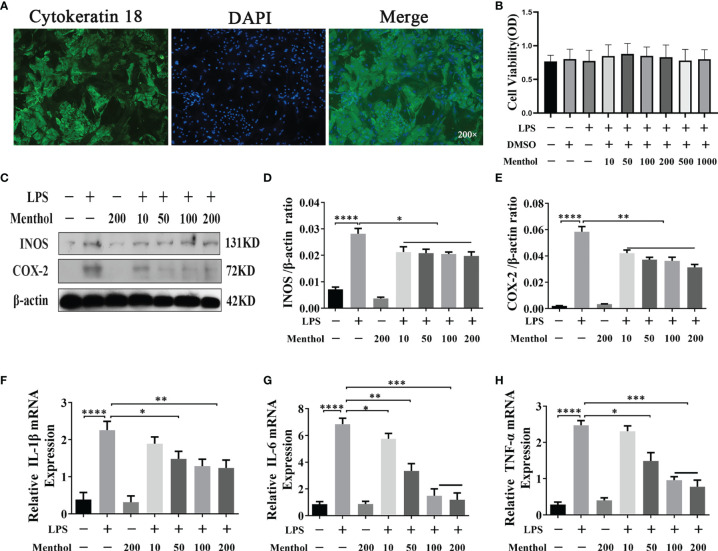
Effect of menthol on the LPS-induced inflammatory response in BMECs. **(A)** Immunofluorescence staining to identify CK-18 expression in BMECs. Nuclei (blue) and CK-18 (green); scale bars are magnification 200x. The BMECs were treated with DMSO (solvent control group) and different concentrations of menthol (10, 50, 100, 200, 500, and 1000 μM) for one hour and then treated with 5 μg/ml LPS for 12 h. **(B)** Results of the cell counting kit (CCK-8) to detect the activity of BMECs (n≥3). BMECs were treated with different concentrations of menthol (10, 50, 100, and 200 μM) for 1 h and LPS at 5 μg/ml was added for 12 h. **(C)** Western blotting assay for INOS and COX-2 protein expression levels (n≥3). **(D, E)** The bar graphs indicate the quantitative results of the corresponding protein bands. **(F–H)** qRT-PCR results for the mRNA levels of IL-6, IL-1β and TNF-α. β-actin was used as an internal reference to normalize the expression levels of protein and mRNA (n≥3). The values are presented as the means ± SEM (*p < 0.05, **p < 0.01, ***p < 0.001 and ****p < 0.0001).

Before exploring the anti-inflammatory effect of menthol, we detected cytokeratin-18 (CK-18) in BMECs using immunofluorescence, which showed strong positive staining for CK-18 (**Figure 1A**), indicating that BMECs have epithelial cell properties. Subsequently, the cytotoxicity of menthol on BMECs was examined using the CCK-8 assay, and the results showed that neither menthol nor LPS caused cytotoxicity (**Figure 1B**). Western blot results showed that LPS significantly upregulated the expression levels of INOS and COX-2, while 10, 50, 100, and 200 μM menthol treatment significantly decreased the expression levels of INOS and COX-2 (**Figures 1C–E**). qRT-PCR results showed that LPS induced overexpression of inflammatory factor-related genes. In contrast, menthol significantly reduced the mRNA expression levels of IL-1β, IL-6 and TNF-α at concentrations of 10, 50, 100, and 200 μM (**Figures 1F–H**). These results suggest that menthol alleviates the inflammatory response of LPS-induced BMECs.

### Menthol Modulates the Effect of LPS on Milk Fat and Milk Protein Expression

**Figure 2:**
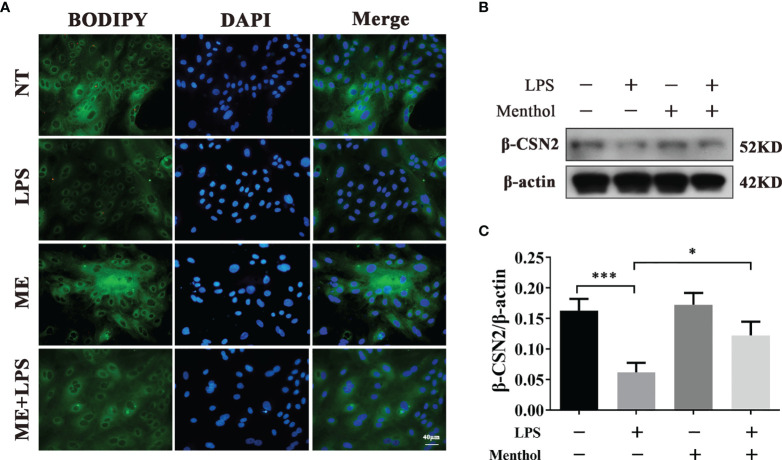
Menthol was able to modulate the effect of LPS on milk lipid and milk protein expression. Menthol at 200 μM was used to treat BMECs for 1 h and then 5 μg/ml LPS was added for 12 h. **(A)** BODIPY staining was used to observe milk fat in BMECs. **(B)** Detection of β-casein expression by Western blotting (n≥3); **(C)** Quantitative results of β-casein bands. β-actin was used as an internal reference to homogenize the protein expression results. The values are presented as the means ± SEM (*p < 0.05 and ***p < 0.001).

Previous studies have shown that LPS stimulation of BMECs leads to a decrease in milk lipid and milk protein expression. To demonstrate whether menthol can mitigate this effect of LPS, we used BODIPY to stain the milk fat in BMECs to observe the milk fat content. The results showed that LPS treatment significantly inhibited the synthesis of milk fat, while menthol pretreatment reversed the downregulation of milk fat synthesis caused by LPS treatment (**Figure 2A**). Additionally, western blot results showed that LPS treatment significantly reduced the expression level of β-casein, while pretreatment with menthol eliminated the inhibitory effect of LPS on β-casein expression (**Figures 2B, C**). The above results suggest that menthol is able to reverse the reduction in milk fat lactoprotein synthesis caused by LPS.

### Menthol Alleviates the LPS-Induced Inflammatory Response by Activating Autophagy in BMECs

**Figure 3:**
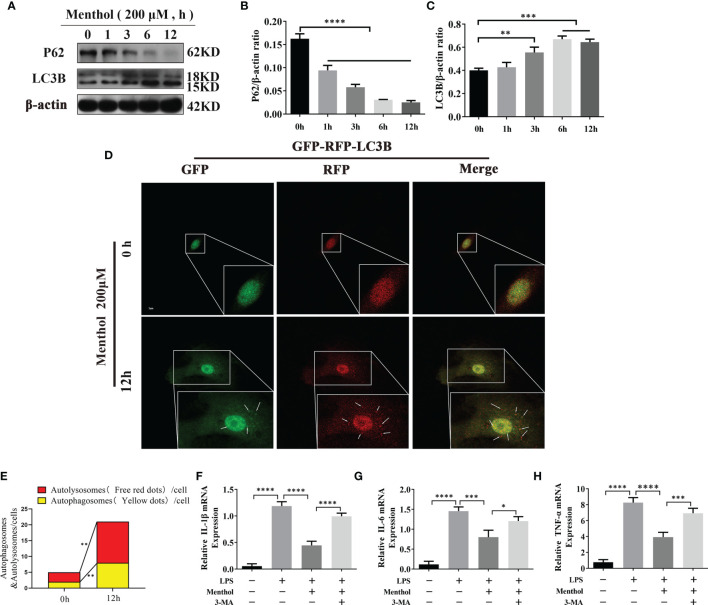
Menthol alleviates the LPS-induced inflammatory response by activating autophagy in BMECs. The BMECs were treated with 200 μM menthol at different time points (0, 1, 3, 6, and 12h). **(A)** Western blotting assay for P62, HO-1, and LC3B protein expression levels (n≥3). **(B, C)** Bar graphs indicate the quantitative results of the corresponding protein bands. β-actin was used as an internal reference to homogenize the protein results. Plasmids carrying mRFP-GFP-LC3B were transfected into BMECs, stably expressed cell were constructed. BMECs treated with 200 μM menthol for different times (0,12h).And images were obtained by confocal microscopy. Yellow spots represent autophagosomes and red spots represent autolysosomes. **(D)** Results of laser confocal detection of expression changes in BMECs of LC3B constructs. **(E)** Results of the quantify of autophagosome and autolysosomes formation, data were obtained from 3 independent experiments, each with a quantitative score of 10 cells. **(F–H)** Results of qRT-PCR for mRNA levels of IL-1β, IL-6, and TNF-α. β-actin was used as an internal reference for homogenization(n≥3). The values are presented as the means ± SEM (*p < 0.05, **p < 0.01, ***p < 0.001 and ****p < 0.0001).

To analyze the effects of menthol on key autophagy proteins at different time points. The results showed that menthol significantly upregulated the expression of LC3B and significantly promoted the degradation of P62 (**Figures 3A–C**). We examined the effect of menthol on autophagic flux by constructing BMECs overexpressing GFP-MRFP-LC3B. The results showed that menthol was able to significantly enhance autophagic flux (**Figures 3D, E**). Then, we inhibited autophagy with 3-MA, an inhibitor of autophagy, and found that the anti-inflammatory effect of menthol was significantly reduced when autophagy was inhibited (**Figures 3F–H**). Therefore, we speculate that menthol may exert its anti-inflammatory effects by inducing the onset of autophagy.

### Menthol Activates AMPK Signaling Pathway

**Figure 4:**
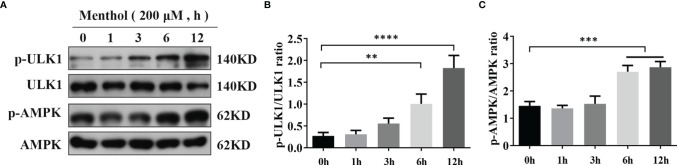
Activation of AMPK pathway by menthol. **(A)** Western blotting assay for p-ULK1, ULK1, p-AMPK, and AMPK protein expression levels (n≥3). **(B, C)** Bar graphs indicate the quantitative results of the corresponding protein bands. The protein expression results were homogenized using β-actin as an internal reference. The values are presented as the means ± SEM (**p < 0.01, ***p < 0.001 and ****p < 0.0001).

Our previous study showed that menthol is able to activate autophagy. To further investigate the pathway of menthol-induced autophagy, we explored the effect of menthol on AMPK. We treated the BMECs with 200 μM menthol at different time points (0, 1, 3, 6, and 12 h). Subsequently, we examined the effect of menthol on AMPK and its downstream related pathways by western blot. The results of the study showed that phosphorylated AMPK expression was significantly increased when menthol was treated for 6h and 12h (**Figures 4A, C**). We also examined the phosphorylation of autophagy-related protein ULK1, and the results showed that menthol treatment could significantly increase the phosphorylation level of ULK1 (**Figures 4A, B**). These findings suggest that the activating effect of menthol on autophagy may act through the activation of AMPK and its downstream related pathways.

### Menthol Promotes Autophagy Through Activation of the AMPK Signaling Pathway

**Figure 5:**
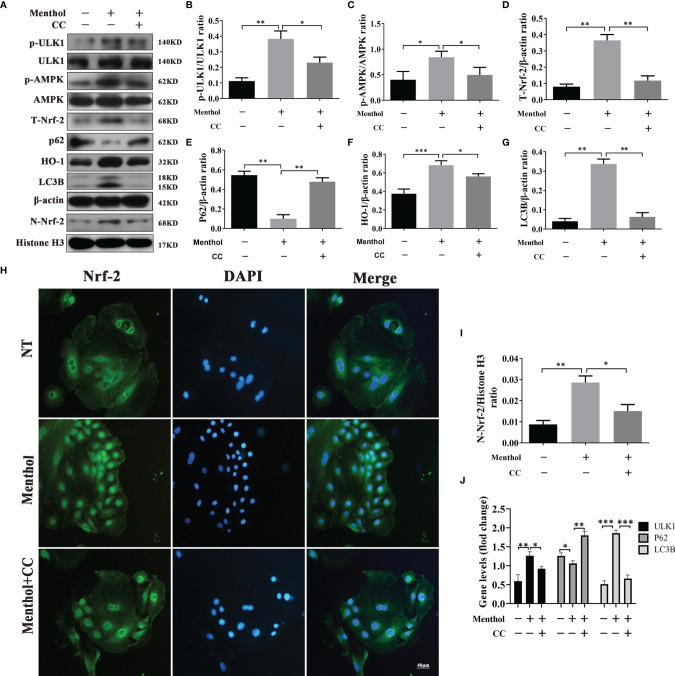
Menthol promotes autophagy through activation of the AMPK/ULK1 or AMPK/Nrf-2 pathways. BMECs were pretreated with 200 μM menthol and CC (inhibitor of AMPK,10 μM) for 1 h and treated with 5 μg/ml LPS for 12 h. **(A)** Protein expression levels of p-ULK1, ULK1, p-AMPK, AMPK, T-Nrf-2, P62, HO-1, LC3B, N-Nrf-2 and Histone H3 were detected by western blotting (n≥3); T-Nrf-2 represents total intracellular Nrf-2, and N-Nrf-2 represents Nrf-2 in the nucleus. **(B)** Detection of Nrf-2 entry by immunofluorescence staining of the nucleus (blue) and Nrf-2 (green). **(C-I)** Bar graphs represent the quantitative results of the corresponding protein bands. The protein results were homogenized using β-actin as an internal reference protein. Histone H3 was used as the internal reference protein in the nucleus. **(J)** Results of qRT-PCR for mRNA levels of ULk1, P62 and LC3B (n≥3). β-actin was used as an internal reference to normalize the mRNA expression. The values are presented as the means ± SEM (*p < 0.05, **p < 0.01 and ∗∗∗p < 0.001).

Previous findings suggest that menthol is able to activate the AMPK pathway. Therefore, we speculate that AMPK may be a key pathway for menthol-induced autophagy. To investigate whether AMPK is associated with menthol-induced autophagy, we inhibited the AMPK signaling pathway using CC (an AMPK inhibitor). The results showed that when AMPK was inhibited, the promotion of p-ULK1, p-AMPK, T-Nrf-2, N-Nrf-2, HO-1 and LC3B protein expression by menthol was also inhibited by western blot assay, while the expression of p62 was significantly upregulated. (**Figures 5A–G, I**). Subsequently, we examined the nucleation of Nrf-2 using immunofluorescence and found that inhibition of AMPK greatly affected the nucleation of Nrf-2 (**Figure 5H**). The results of qRT-PCR also showed that the mRNA levels of ULK1 and LC3B were significantly lower in the menthol+CC group than in the menthol group, while the mRNA levels of P62 were significantly higher than in the menthol group (**Figure 5J**). These results suggest that menthol is able to induce autophagy through activation of the AMPK/ULK1 and AMPK/Nrf-2 pathway.

### Menthol Alleviates the Inflammatory Response in BMECs Through the AMPK Signaling Pathway

**Figure 6:**
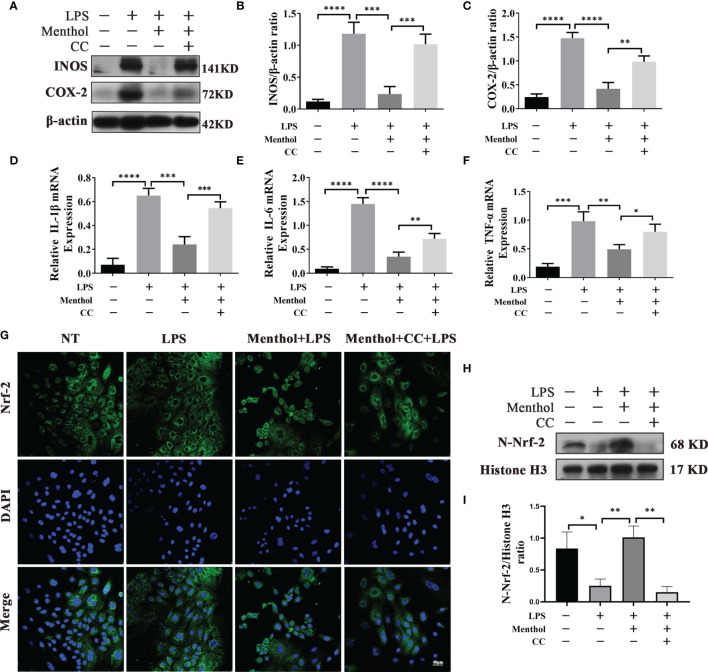
Menthol alleviates the inflammatory response of BMECs through the AMPK signaling pathway. BMECs were pretreated with 200 μM menthol and CC for 1h and treated with 5 μg/ml LPS for 12 h. **(A)** Protein expression results of INOS and COX-2 detected by western blotting(n≥3). **(B, C)** Bar graphs indicate the quantitative results of the corresponding protein bands. β-actin was used as an internal reference protein to homogenize the protein results. **(D–F)** Results of qRT-PCR for mRNA levels of IL-1β, IL-6, and TNF-α. β-actin was used as an internal reference for normalization of mRNA expression(n≥3). **(G)** Detection of Nrf-2 entry by immunofluorescence staining of the nucleus (blue) and Nrf-2 (green). **(H)** Protein expression results of N-Nrf-2 and Histone H3 detected by western blotting(n≥3). **(I)** Bar graphs indicate the quantitative results of the corresponding protein bands.Histone H3 was used as an internal reference protein to homogenize the protein results. The values are presented as the means ± SEM (*p < 0.05, **p < 0.01, ***p < 0.001 and ****p < 0.0001).

In our previous findings, we found that menthol can activate autophagy by activating AMPK and its downstream pathways. To investigate the key role of AMPK in the process of anti-inflammatory effect of menthol., We used CC treatment of menthol-treated LPS-induced inflammation models of BMECs. Western blot results showed that menthol significantly inhibited the expression of INOS and COX-2, while the down-regulation of INOS and COX-2 by menthol was significantly suppressed after inhibition of AMPK using CC (**Figures 6A–C**) Subsequently, we examined the mRNA of inflammatory factors using qRT-PCR technique. The results suggested that menthol was able to significantly down-regulate RNA expression of IL-6, IL-1β and TNF-α, and this down-regulation by menthol was inhibited after CC treatment (**Figures 6D–F**). In addition, we examined the effect of menthol on the situation of Nrf-2 into the nucleus by immunofluorescence technique and western blot. The results of immunofluorescence assay showed that menthol could significantly promote the entry of Nrf-2 into the nucleus. And the nucleus entry of Nrf-2 was significantly inhibited after treatment with CC (**Figure 6G**). The results of western blot were consistent with the results of immunofluorescence (**Figures 6H, I**). The above results suggest that menthol can inhibit LPS-induced inflammatory responses in BMECs by activating autophagy through the AMPK signaling pathway.

### Menthol Promotes Autophagy in BMECs by Activating the Nrf-2/HO-1 Signaling Pathway

**Figure 7:**
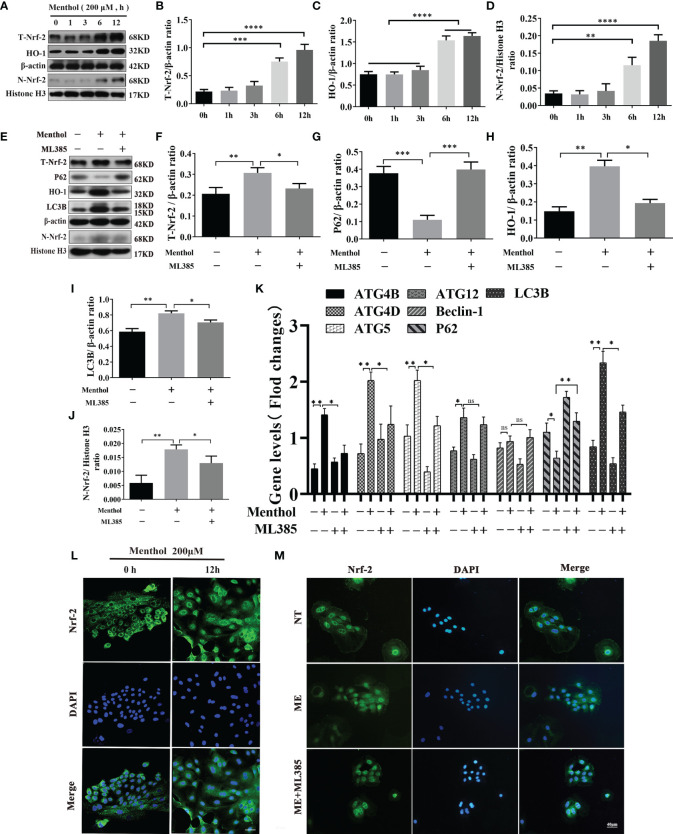
Menthol can promote the expression of autophagy-related genes by activating the Nrf-2/HO-1 signaling axis. The BMECs were treated with 200 μM menthol at different time points (0, 1, 3, 6, and 12h). **(A)** Western blotting for the protein expression levels of T-Nrf-2, HO-1, N-Nrf-2 and Histone H3 (n≥3); T-Nrf-2 represents total intracellular Nrf-2 and N-Nrf-2 represents Nrf-2 in the nucleus. **(B–D)** Bar graphs indicate the quantitative results of the corresponding protein bands. Then 200 μM menthol and ML385 (inhibitor of Nrf-2, 5 μg/ml) were pretreated with bovine mammary epithelial cells for 1 h. LPS at 5 μg/ml was added for 12 h. **(E)** Protein expression levels of T-Nrf-2, p62, HO-1, LC3B, N-Nrf-2 and Histone H3 were detected by western blotting (n≥3); T-Nrf-2 represents total intracellular Nrf-2, and N-Nrf-2 represents Nrf-2 in the nucleus. **(F–J)** The bar graph indicates the quantitative results of the corresponding protein bands. β-actin was used as an internal reference protein to homogenize the protein results. Histone H3 was used as the internal reference protein in the nucleus. **(K)** Results of qRT-PCR assay for mRNA levels of ATG4B, ATG4D, ATG5, ATG12, Beclin-1, P62, LC3B (n≥3). The mRNA expression of β-actin was used to normalize the mRNA expression of the above proteins. The BMECs were treated with 200 μM menthol at different time points (0, and 12 h). **(L)** Immunofluorescence staining to detect the entry of Nrf-2 into the nucleus, nucleus (blue), and Nrf-2 (green). Then 200 μM menthol and ML385 (inhibitor of Nrf-2, 5 μg/ml) were pretreated with bovine mammary epithelial cells for 1 h LPS at 5 μg/ml was added for 12 h. **(M)** Immunofluorescence staining to detect the entry of Nrf-2 into the nucleus, nucleus (blue), and Nrf-2 (green). The values are presented as the means ± SEM (ns means no difference, *p < 0.05, **p < 0.01, ***p < 0.001, and ****p < 0.0001).

Autophagy activation involves multiple pathways, and the pathway of menthol-induced autophagy was further investigated. We treated BMECs with 200 μM menthol at different time points (0, 1, 3, 6, and 12 h). Western blot results showed that menthol was able to significantly upregulate intracellular total Nrf-2 expression when the treatment time was at 6h and 12h (**Figures 7A, B**). And menthol treatment also significantly up-regulated the amount of Nrf-2 in the nucleus and promoted the entry of Nrf-2 into the nucleus(**Figures 7A, D**). Also, our results revealed that menthol significantly increased HO-1 expression (**Figures 7A, C**). Subsequently, we detected the intracellular distribution of Nrf-2 by immunofluorescence technique. The results showed that the distribution of Nrf-2 in the nucleus was increased when BMECs were treated with menthol for 12 h, suggesting that menthol could significantly promote the nuclear entry of Nrf-2. (**Figure 7L**). To further investigate the key role of Nrf-2 in menthol-activated autophagy, we added ML385 (an inhibitor of Nrf-2). Western blot results showed that menthol significantly increased intracellular total Nrf-2 and intracellular Nrf-2, while ML385 was able to significantly inhibit this change (**Figures 7E, F, J**). Meanwhile, treatment with menthol significantly upregulated the expression of HO-1, while treatment with ML385 significantly inhibited the effect of menthol (**Figure 7H**). In addition, the upregulation of the autophagy marker LC3B by menthol and the downregulation of P62 were inhibited by ML385(**Figures 7E, G, I**). Subsequently, we examined the effect of menthol or ML385 on autophagy-related genes by qRT-PCR.The results also showed that inhibition of Nrf-2 significantly reduced the expression of most autophagy-related genes. Interestingly, we found that menthol activation of ATG12 expression was Nrf-2 independent and that menthol had no effect on Beclin-1 mRNA expression. (**Figure 7K**). The immunofluorescence results were also consistent with the western blot results (**Figure 7M**). The above results suggest that menthol is able to promote autophagy by activating the Nrf-2 pathway.

### Menthol Alleviates the Inflammatory Response Through the Nrf-2/HO-1 Signaling Axis

**Figure 8:**
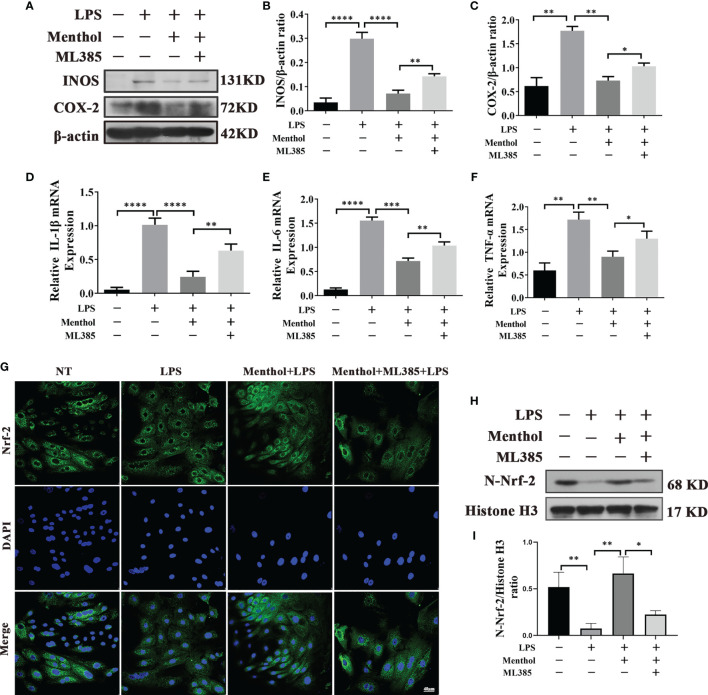
Menthol alleviates the inflammatory response through the Nrf-2/HO-1 signaling axis. The BMECs were first pretreated with 200 μM menthol and ML385 for 1 h. LPS at 5 μg/ml was added for 12 h. **(A)** Protein expression results of INOS and COX-2 detected by western blotting (n≥3). **(B, C)** The bar graphs indicate the quantitative results of the corresponding protein bands. The protein results were homogenized using β-actin as an internal reference protein. **(D–F)** Results of qRT-PCR to detect the mRNA levels of IL-1Bβ, IL-6, and TNF-α (n≥3). The mRNA expression of β-actin was used to normalize the mRNA expression of the above proteins. **(G)** Immunofluorescence staining to detect the entry of Nrf-2 into the nucleus, nucleus (blue), and Nrf-2 (green). **(H)** Protein expression results of N-Nrf-2 and Histone H3 detected by western blotting(n≥3). **(I)** Bar graphs indicate the quantitative results of the corresponding protein bands.Histone H3 was used as an internal reference protein to homogenize the protein results. The values are presented as the means ± SEM (*p < 0.05, **p < 0.01, ***p < 0.001 and ****p < 0.0001).

Our study found that the Nrf-2/HO-1 signaling axis has an important role in maintaining the activation of autophagy. Although Nrf-2 plays a key role in maintaining the autophagy activated by menthol. Does Nrf-2 also play an important role in menthol anti-inflammation? Subsequently, we conducted the following study. We used ML385 and/or menthol treatment in a model of LPS-induced inflammation in BMECs. Western blot results showed that Menthol significantly inhibited the expression of INOS and COX-2, while the down-regulation of INOS and COX-2 by menthol was significantly suppressed after AMPK inhibition using ML385 (**Figures 8A–C**). Meanwhile, the results of qRT-PCR showed that treatment with ML385 significantly inhibited the down-regulation of RNA expression of IL-6, IL-1β and TNF-α by menthol (**Figures 8D–F**). In addition, we also examined the effect of menthol on Nrf-2 entry into the nucleus by immunofluorescence technique and western blot. And the immunofluorescence results showed that menthol can increase the distribution of Nrf-2 in the nucleus. In contrast, the distribution of Nrf-2 in the nucleus was significantly diminished after treatment with ML385 The results of western blot were consistent with those of immunofluorescence, and treatment with ML385(**Figure 8G**). significantly down-regulated the amount of Nrf-2 protein in the nucleus(**Figures 8H, I**).The above results suggest that menthol can inhibit LPS-induced inflammatory responses in BMECs by activating autophagy through the Nrf-2 pathway.

## Discussion

**Figure 9:**
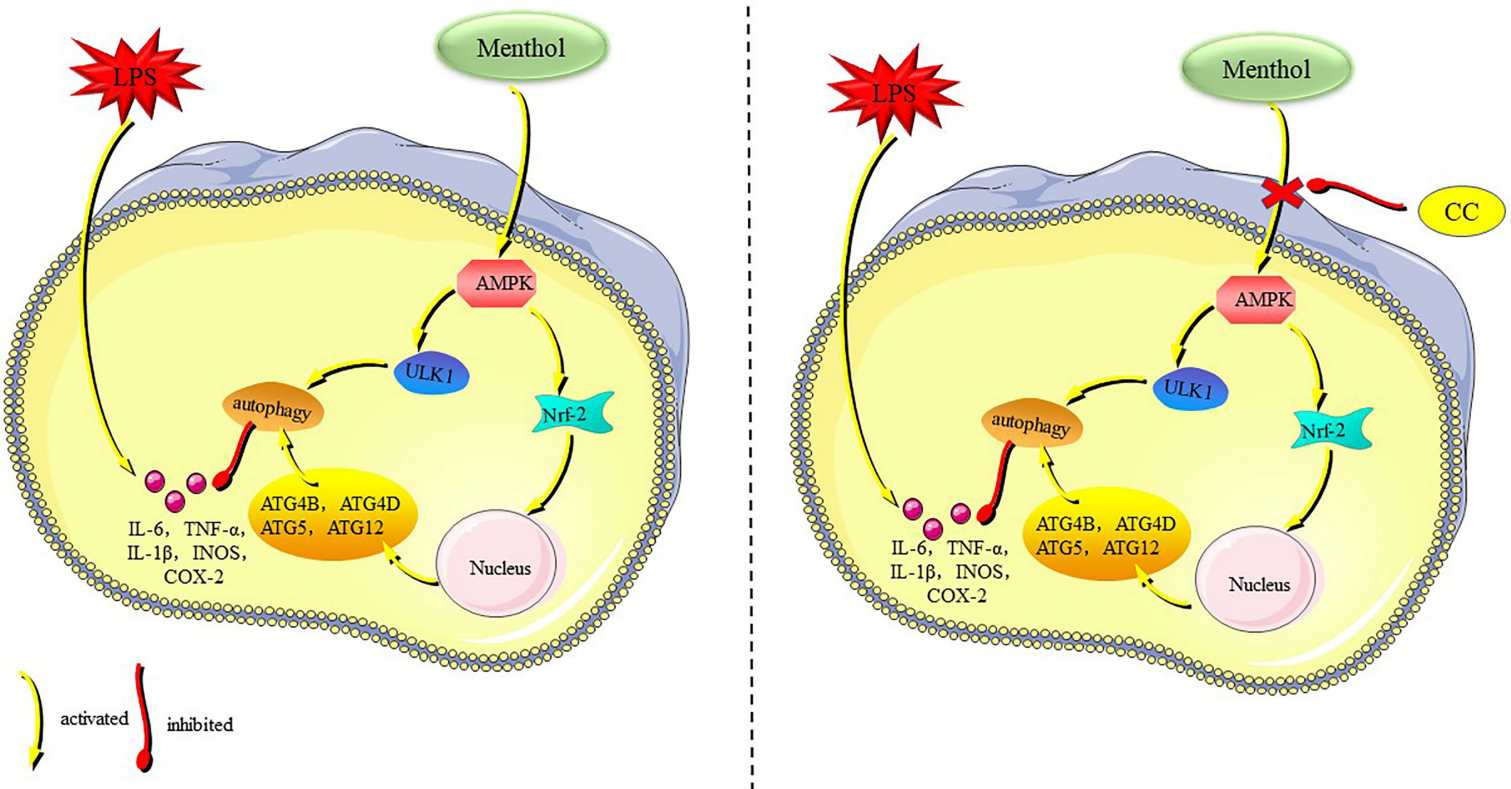
The mechanism of menthol in anti-inflammatory.

Menthol is a terpenoid with a variety of biological activities, and numerous studies have shown that menthol has significant anti-inflammatory effects (21, 29, 30). Our results showed that menthol significantly attenuated the inflammatory response in LPS-induced BMECs, and the main mechanism of action was that menthol promoted autophagy through activation of the AMPK/ULK1 pathway and AMPK/Nrf-2 pathway, which in turn inhibited the expression of inflammatory factors and proinflammatory enzymes (**Figure 9**). In this study, we demonstrated the ability of menthol to protect BMECs from damage caused by inflammatory responses through the induction of autophagy.

Mastitis is a complex mammary syndrome caused by a conditionally pathogenic bacterium that manifests itself as an invasion of the mammary gland by the pathogen and triggers a violent inflammatory response (31). BMECs in dairy cows are the most important functional cells in mammary tissue, with important roles in the secretion and synthesis of milk and protection against invading pathogenic microorganisms. Jenean et al. found that BMECs are the first to come into contact with exogenous microorganisms, recognize and respond, and function similarly to sentinel cells, performing immune surveillance functions when bacteria invade (32). BMECs not only respond to pathogenic bacteria, but also recruit other immune cells in preparation for further elimination of pathogens (10, 33). LPS is a cell wall component of gram-negative bacteria and a major model molecule causing mammary inflammation (31, 34). Our study found that LPS stimulation of BMECs significantly upregulated inflammatory factor levels, but menthol pretreatment significantly inhibited the secretion of LPS-induced inflammatory factors, suggesting a protective effect of menthol during the inflammatory response of BMECs. In addition, BMECs, as one of the main cells of mammary tissue, are not only important immune cells, but also a major cell population for lactation in cows (28, 35). When mastitis occurs in cows, mastitis is well known to seriously affect milk production and quality (11, 19, 34). Yusaku Tsugami et al. found that IL-1β decreased β-casein and triglyceride secretion, and tumor necrosis factor-α inhibited β-casein secretion, suggesting that inflammatory factors may be the main cause of reduced milk lipid and milk protein expression (31). Interestingly, we found a protective effect of menthol on the lactation function of BMECs by assessing the expression levels of triglycerides and β-casein. These results suggest that menthol may restore lactation function in BMECs by inhibiting the expression of inflammatory factors. Although menthol is able to protect BMECs by suppressing the expression of inflammatory factors, its specific mechanism of action has not been reported.

AMPK acts as an energy sensor in the cell to sensitively display changes in energy in the cell and is a key regulatory molecule of energy metabolism in the cell (36). Numerous studies have found that activation of AMPK helps to improve cell survival in inflammatory states and attenuates the inflammatory response (37–39). And, we found that the anti-inflammatory effect of menthol was significantly affected when AMPK was inhibited, and the inhibitory effect on inflammatory factors is significantly reduced. Rahman et al. found that resveratrol protects neurons from damage caused by inflammatory responses through activation of the AMPK pathway (38). This protection from damage is consistent with our findings, suggesting that menthol may exert biological functions such as anti-inflammatory and antioxidant functions through the AMPK pathway. It is well known that autophagy is an important process for maintaining cellular homeostasis, which is highly conserved in eukaryotic cells and regulated by autophagy genes (15, 16, 40). Numerous studies have found that autophagy has a very important role in maintaining inflammatory homeostasis, eliminating pathogenic bacterial invasion and participating in the innate immune response (41–43). A study by Wang Z. et al. found that taurine was able to attenuate staphylococcal-induced inflammatory damage by activating autophagy in BMECs (44). Related reports suggest that there are classical and nonclassical pathways for the activation of autophagy. In contrast, the nonclassical pathway of autophagy is mainly mediated by the AMPK pathway. Therefore, we hypothesized that the anti-inflammatory effect of menthol may be related to the activation of the nonclassical pathway of autophagy, which is dependent on the activation of AMPK.

Our previous study found that menthol significantly activated the AMPK signaling pathway, so we speculate that it may also affect ULK1 and Nrf-2 autophagy-related pathways (39, 41). ULK1 is a downstream protein of AMPK and is able to induce autophagy when ULK1 is activated. Numerous studies have shown that the AMPK-ULK1 pathway is essential for the regulation and induction of autophagy, and AMPK can directly phosphorylate ULK1 and thus promote autophagy (38, 39, 45). Wei Xiong et al. found that UCP1 was able to activate autophagy through the AMPK/ULK1/autophagy pathway and attenuate acute kidney injury triggered by lipid accumulation (36). Interestingly, our findings revealed that menthol is also able to promote autophagy by remembering the AMPK/ULK1 pathway. When the AMPK pathway is blocked, not only is autophagy inhibited, but the expression of inflammatory factors is also significantly increased, implying that menthol may exert anti-inflammatory effects by inducing autophagy through the AMPK/ULK1 pathway. The nuclear transcription factor Nrf-2 is considered an important regulator of cellular homeostasis; and is capable of activating the expression of a variety of cytoprotective genes (46). AMPK, as a kinase working upstream of Nrf-2, has an important role in the regulation of Nrf-2 (19). Under normal conditions, Nrf-2 exists in free form, in the cytoplasm. When cells are subjected to inflammatory stimuli or redox imbalance, the upstream AMPK protein is activated, which in turn promotes the entry of free Nrf-2 into the nucleus and activates the expression of downstream related genes (47). Previous studies have shown that Nrf-2 is a key regulatory protein of autophagy-related genes (48). And, our study found significant downregulation of autophagy-related genes after inhibition of Nrf-2, which is consistent with previous studies. Interestingly, we also identified some autophagy-related genes that are not regulated by Nrf-2, but these genes may not be involved in menthol-mediated autophagic processes. We also found that inhibition of Nrf-2 resulted in a diminished anti-inflammatory effect of mentholatum. Therefore, we suggest that the activation of Nrf-2 can play a role in promoting autophagy activation in concert with ULK1. The Nrf-2 pathway is also able to exert some anti-inflammatory functions directly.

Based on the above findings we found that menthol could maintain the autophagic response activated by AMPK/ULK1 through activation of AMPK/Nrf-2 and thus exert anti-inflammatory effects.

## Conclusion

Menthol protects BMECs against LPS-induced inflammatory damage by decreasing the expression of proinflammatory mediators. Menthol upregulates LC3B, downregulates P62 and promotes the transcription of autophagy-related genes, suggesting that autophagy is involved in the cytoprotective effects of menthol. In addition, menthol was able to upregulate AMPK and ULK1 phosphorylation and increase the nuclear localization of Nrf-2, suggesting that menthol may exert its anti-inflammatory effects through the AMPK/ULK1/Nrf-2/autophagy pathway. Notably, autophagy was significantly inhibited when the AMPK pathway was blocked, whereas autophagy levels were significantly decreased when the Nrf-2 pathway was blocked. In conclusion, autophagy plays a crucial role in the process by which menthol protects against inflammatory injury in BMECs.

## Data Availability Statement

The original contributions presented in the study are included in the article/supplementary material. Further inquiries can be directed to the corresponding author.

## Author Contributions

SQL, GH, and WG designed the study. SQL, WG, and SL performed the experiments. SQL, BY, and JL analyzed the data. WG, SQL, and GH contributed materials and tools. SQL, SF, YJ, GH, and WG prepared the manuscript. All authors reviewed and approved the manuscript.

## Funding

This work was supported by the National Natural Science Foundation of China. Grant/Award Numbers: 31873004, 32172807, 32102624; Jilin Young Scientific and Technological Talents Promotion Project: QT202127.

## Conflict of Interest

The authors declare that the research was conducted in the absence of any commercial or financial relationships that could be construed as a potential conflict of interest.

## Publisher’s Note

All claims expressed in this article are solely those of the authors and do not necessarily represent those of their affiliated organizations, or those of the publisher, the editors and the reviewers. Any product that may be evaluated in this article, or claim that may be made by its manufacturer, is not guaranteed or endorsed by the publisher.
